# How to choose treatment for patients who are eligible for both unicompartmental knee arthroplasty and high tibial osteotomy?

**DOI:** 10.1016/j.jot.2025.10.015

**Published:** 2025-12-22

**Authors:** Jianbin Guo, Zhibo Liu, Yong Ding

**Affiliations:** a Honghui Hospital, Xi'an Jiaotong University, Xi'an, 710054, China; b Xi'an Key Laboratory of Arthritis Pathogenesis and Precision Treatment, Xi'an, 710054, China

**Keywords:** High tibial osteotomy, Indication standards, Preoperative factors, Surgical decision-making, Unicompartmental arthritis, Unicompartmental knee arthroplasty

## Abstract

Osteoarthritis (OA) is highly prevalent among older adults, with most cases beginning as early unicompartmental arthritis. Surgical treatments, such as unicompartmental knee arthroplasty (UKA) or high tibial osteotomy (HTO), are chosen based on the patient's intra-articular or extra-articular deformity. However, there are overlapping ‘gray areas’ in the classic indications for these two surgical methods, making the decision between these two procedures less straightforward.

Several recent studies have underscored the importance of refining these indications. For example, multiple long-term studies have demonstrated the durability of UKA in well-selected patients, while others suggest that HTO may offer superior functional outcomes in younger, active individuals. Research has increasingly focused on identifying key preoperative factors that could better predict the success of these procedures. For instance, preoperative exercise intensity, which reflects the patient's physical activity levels and joint adaptability, has been shown to significantly influence post-surgical outcomes for both UKA and HTO.

This study aims to bridge the current gap in surgical decision-making by assessing a range of preoperative factors, including exercise intensity, meniscal status, anatomical structure, soft tissue laxity, bone marrow edema, spontaneous insufficiency fractures of the knee, osteoporosis, as well as basic demographic data such as gender, age, and weight. The goal is to develop more robust and scientifically grounded criteria for determining the most appropriate surgical treatment particularly for patients in the ‘gray areas’ of indications. By establishing these more refined standards, orthopedic surgeons will be better positioned to optimize patient outcomes and reduce the risk of revision surgeries.

The Translational Potential of this Article: This study systematically evaluated a range of preoperative factors to enhance surgical decision-making for patients who fall within the “gray areas” of UKA and HTO indications. By refining patient selection, this approach aims to improve individualized treatment, optimize postoperative functional recovery, and reduce revision rates. In addition, it seeks to establish a scalable predictive model and decision-making framework, offering evidence-based support for the standardization and refinement of surgical strategies in osteoarthritis.

## Graphical abstract

**Figure:**
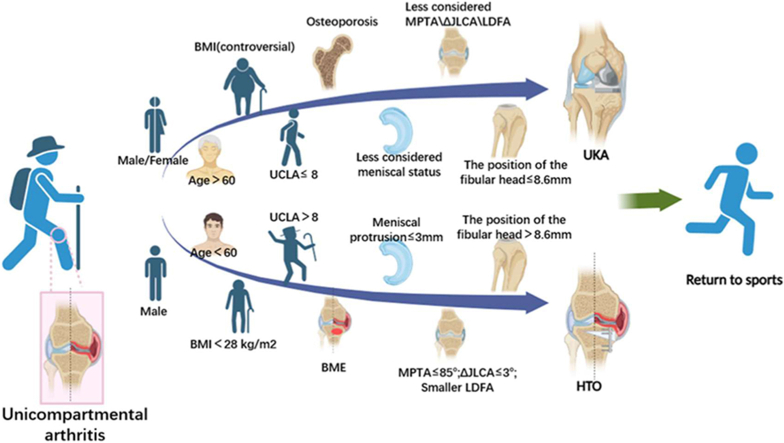

## 1 Introduction

Osteoarthritis (OA) is a degenerative disease that affects millions of people annually. One of the most common forms of this condition is knee osteoarthritis [1,2]. About one-third of OA cases manifest as unicompartmental arthritis. Depending on the patient's age, activity level, and the clinical characteristics of the knee joint, several surgical methods have been proposed to address it. These methods include high tibial osteotomy (HTO), unicompartmental knee arthroplasty (UKA), and total knee arthroplasty (TKA) [3,4]. HTO and UKA are preferred in the early stages of the disease as they preserve more of the original knee joint structure, often delaying the need for final TKA treatment. HTO serves as a treatment for OA associated with knee joint dislocation. It effectively alleviates knee pain and decelerates OA progression by realigning the mechanical axis of the knee joint from the arthritic compartment to a neutral position. Traditionally, HTO is considered ideal for young, active, non-obese patients with isolated knee varum deformity resulting from medial compartment OA [5,6]. For UKA, the current classic indications include: unicompartmental arthritis, absence of anterior or posterior cruciate ligament injury, a range of motion of at least 90°, minimalvarus deformity (<5°), minimalflexion deformity (<10°) and absence of obesity [3,7].

**Fig. 1:**
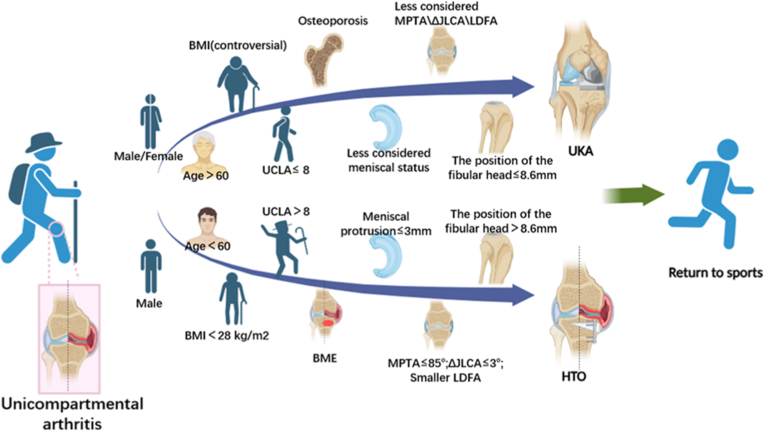
Facilitate more precise clinical decisions for patients with unicompartmental arthritis. Body mass index (BMI); University of California and Los Angeles activity (UCLA); Bone marrow edema (BME); Medial proximal tibial angle (MPTA); Joint line convergence angle (JLCA); The disparity in JLCA between supine radiographs and standing radiographs (ΔJLCA); lateral distal femoral angle (LDFA); The distance from the tibial articular surface to the tip of the fibular head (The position of the fibular head).

In clinical practice, there remains controversy regarding whether patients with unicompartmental arthritis should opt for UKA or HTO for better prognosis [8,9]. This article aims to address this uncertainty by reviewing various factors that could impact the prognosis after HTO and UKA. These factors include preoperative exercise intensity, preoperative meniscal status, preoperative anatomical angle, soft tissue laxity, preoperative bone marrow edema, spontaneous insufficiency fracture of the knee, osteoporosis, as well as gender, age, and weight. By considering these preoperative factors, the article aims to offer insights for making more informed treatment decisions for patients who meet the traditional indications for both UKA and HTO.(see.fig. 1)

### 1.1 Preoperative exercise intensity

Current research focuses on comparing the outcomes of unicompartmental knee arthroplasty (UKA) and high tibial osteotomy (HTO). The findings suggest that both procedures yield positive results [10,11]. Overall, HTO is generally recommended for patients with higher activity levels, while UKA is more suitable for older patients [12] and few studies have compared the efficacy of these two surgical methods based on patients' preoperative exercise levels. Christophe Jacquet et al. found that in a group of patients with a pre-arthritic University of California and Los Angeles activity (UCLA) score>8, those treated with HTO could return to sports activities and previous professional activities faster than those treated with UKA. Additionally, they had higher motor-related function scores two years postoperatively [9,13]. Some studies have also pointed out that continued participation in sports before surgery is more conducive to returning to sports after HTO [14]. In addition, studies have shown that approximately two-thirds of patients who underwent HTO returned to sports and work within one year after surgery, with their level of sports participation being equal to or greater than their preoperative level [15]. Therefore, patients who meet the indications for both HTO and UKA and engage in high-intensity exercise before surgery may be more suitable candidates for HTO surgical treatment.

### 1.2 Preoperative meniscal status

**Fig. 2:**
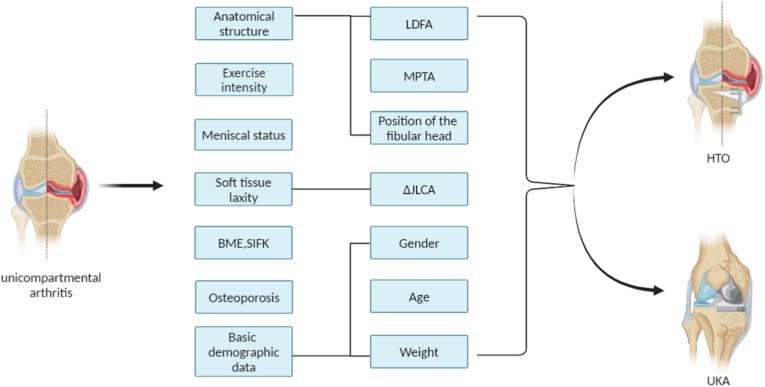
Recent studies have shown that for patients with unicompartmental arthritis who meet the classical indications for both high tibial osteotomy (HTO) and unicompartmental knee arthroplasty (UKA), various factors such as anatomical structure, exercise intensity, meniscal status, soft tissue laxity, bone marrow edema (BME), spontaneous insufficiency fracture of the knee (SIFK), osteoporosis, and basic demographic data should be evaluated to determine the most suitable treatment.

The medial meniscus plays a crucial role in knee joint stability, lubrication, load-bearing, and impact resistance [16]. When the meniscal collagen fibers rupture, the meniscus can protrude outside the tibial plateau. This damage weakens the meniscus's ability to maintain proper knee joint movement and accelerates the progression of OA [17]. Recent studies have found that the severity of preoperative medial meniscal extrusion is closely related to joint pain 1–2 years after medial opening wedge high tibial osteotomy (MOWHTO) [18]. Hong-Yeol Yang et al. found that patients with medial meniscus extrusion > 3 mm before HTO surgery experienced more significant postoperative pain than those with extrusion < 3 mm [19]. In addition to the impact of preoperative meniscal extrusion on the efficacy of HTO, Chang-Hyun Lee et al. found that patients with medial meniscus extrusion < 3 mm after HTO surgery had better clinical outcomes than those with extrusion > 3 mm. Furthermore, the improvement in medial meniscus extrusion after MOWHTO is related to the preoperative varus alignment of the lower limbs [20]. Further studies have found that repairing the medial meniscus posterior root can enhance the biomechanical structure and significantly improve postoperative outcomes in patients undergoing HTO surgery [21]. Therefore, before patients undergo HTO surgery, their medial meniscal extrusion should be considered to select the most appropriate treatment method. To improve the prognosis of HTO surgery, a study with a one-year follow-up found that combining HTO with the repair of medial meniscus posterior root tears (MMPRTs) can promote meniscal healing and regeneration of the medial femoral condyle cartilage. However, the short-term clinical results one year after surgery were not significantly different from those treated with HTO alone [22]. Another study with a two-year follow-up also found that combining MMPRT repair with HTO did not provide significant clinical benefits, aside from increasing the rate of meniscal healing [23]. A retrospective study with a mean follow-up of 30 months found that HTO combined with MMPRT resulted in better clinical outcomes and higher return-to-sports rates compared to HTO alone [24]. The existence of this controversy suggests that, compared to HTO alone, the benefits of combining MMPRT repair with HTO may become more apparent in the mid-to long-term postoperative period [25]. However, UKA surgery involves the direct removal of the medial meniscus. Therefore, considering the preoperative condition of the meniscus, UKA offers a broader range of indications (see Fig. 2).

### 1.3 Preoperative anatomical structure

**Fig. 3:**
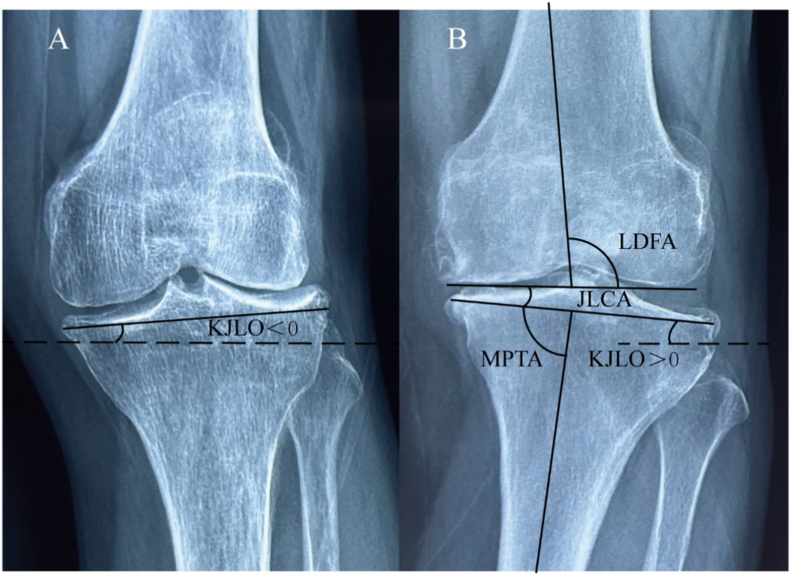
Anatomical structure angles of the knee joint. Lateral distal femoral angle (LDFA); Joint line convergence angle (JLCA); Knee joint-line obliquity (KJLO); Medial proximal tibial angle (MPTA).

Knee joint-line obliquity (KJLO). KJLO refers to the angle in the coronal plane between a horizontal line and a straight line tangent to the articular surface of the proximal tibia. Postoperative KJLO is closely linked to the prognosis of HTO [26,27]. Nakayama H et al. have revealed that a valgus angle of 5–10° in KJLO increases the shear stress on articular cartilage following HTO [28]. Ju-Ho Song et al. highlighted that when KJLO is ≥ 6°, it adversely affects the prognosis of HTO surgery, suggesting the consideration of appropriate treatment methods [29]. To delve deeper into the factors impacting postoperative KJLO, certain studies have revealed that preoperative lateral distal femoral angle (LDFA) positively correlates with postoperative KJLO after HTO, serving as a significant predictor for it [30] (Fig. 3). Therefore, patients with an excessively high preoperative LDFA should carefully consider HTO surgery. However, since UKA primarily addresses intra-articular deformities, there are currently no studies demonstrating the impact of LDFA on its prognosis. Based on existing research, the recommended treatment methods for patients with unicompartmental arthritis, considering preoperative LDFA and JLCA, are presented in (Table 2). In addition, Tae Woo Kim et al. found that a high fibula position before HTO surgery is associated with a higher incidence of lateral hinge fracture post-surgery [31]. Further studies have identified critical values for the position of the hinge (The distance from the tibial articular surface to the hinge point) and fibular head (The distance from the tibial articular surface to the tip of the fibular head), which are 13.3 mm and 8.6 mm, respectively. When the hinge (<13.3 mm) or fibular head (<8.6 mm) is positioned excessively high, the likelihood of a lateral hinge fracture increases significantly [32,33]. For UKA, there is currently no research indicating that a higher fibular head affects surgical outcomes. Therefore, we currently recommend UKA treatment for patients with a high fibular head position before surgery. The impact of the medial proximal tibial angle (MPTA) on the prognosis of HTO surgery is significant and merits attention [34,35]. A retrospective study with over two years of follow-up reported that patients with a pre-HTO MPTA≥85° had worse clinical outcomes in terms of functional scores compared to those with a pre-HTO MPTA<85° [36]. The impact of changes in the MPTA on patient prognosis after HTO surgery also deserves attention. A study with a 4-year follow-up indicated that an increase in MPTA to greater than 95° after HTO did not significantly affect the patient's clinical outcome or the deterioration of lateral compartment cartilage. However, lateral compartment pain was significantly more frequent in these patients [37]. Some studies have also pointed out that an excessively elevated MPTA after HTO has no significant impact on short-term clinical performance but can lead to ACL degeneration [38]. A retrospective study with a mean follow-up time of five years found that patients with a preoperative MPTA greater than 80° had no significant difference in clinical prognosis after UKA compared to patients with an MPTA less than 80° [39]. This indicates that patients with a preoperative MPTA greater than 85° should be more cautious when considering HTO treatment options. Therefore, we recommend UKA treatment for patients with a large preoperative MPTA, though this suggestion needs to be confirmed by further research.

**Table 1:** Factors affecting the prognosis of UKA or HTO surgery.

Factors	UKA	HTO	Reference
Gender	Gender does not have a significant relationship with patient imaging results or clinical manifestations.	Men are more suitable candidates, while women present a higher risk factor for postoperative revision.	[61,62]
Age	The grouping of nodes at different ages does not have a significant relationship with prognosis.	Patients under 60 years old had lower revision and complication rates	[63,65]
Weight	There is controversy over whether obesity contributes to poor surgical outcomes.	BMI ≥28 kg/m^2^ is an risk factor for deep vein thrombosis following surgery.	[73,77,78]
Preoperative exercise intensity	Patients with high preoperative exercise levels take longer to return to exercise post-surgery.	Patients with a preoperative ULCA score greater than 8 return to sports faster.	[9,14]
Meniscal status	Direct surgical removal of the meniscus	A protrusion greater than 3 mm exacerbates postoperative pain.	[18,19]
Postoperative KJLO	Surgery does not significantly alter KJLO scores.	KJLO≥6° is associated with a poor prognosis.	[28,29]
Preoperative LDFA	Surgery does not significantly alter LDFA scores.	When preoperative LDFA ≥88°, there is a risk of postoperative KJLO >5°.	[30]
MPTA	No significant impact on surgical prognosis.	Preoperative temperature ≥85° leads to adverse clinical prognosis	[36,39]
ΔJLCA	Surgery does not significantly alter ΔJLCA scores.	ΔJLCA>3° leads to overcorrection.	[45]
Osteoporosis	No significant impact on the prognosis of tibial component migration or pain after surgery.	A risk factor for requiring subsequent revision surgery.	[59,60]
BME	Related to postoperative pain	No significant impact on surgical prognosis.	[49,51]
The position of the fibular head	No significant impact on surgical prognosis.	The critical value is 8.6 mm. If it is lower than this, it may more easily cause a hinge fracture.	[31,32]

**Table 2:** Different preoperative JLCA and LDFA have a predictive effect on postoperative KJLO. When KJLO>5°, UKA is more recommended, and conversely, HTO treatment is more recommended.

		LDFA										
		85	86	87	88	89	90	91	92	93	94	95
JLCA	−1	HTO	HTO	HTO	HTO	HTO	HTO	HTO	HTO	HTO	HTO	UKA
	0	HTO	HTO	HTO	HTO	HTO	HTO	HTO	HTO	HTO	UKA	UKA
	1	HTO	HTO	HTO	HTO	HTO	HTO	HTO	HTO	UKA	UKA	UKA
	2	HTO	HTO	HTO	HTO	HTO	HTO	HTO	UKA	UKA	UKA	UKA
	3	HTO	HTO	HTO	HTO	HTO	HTO	UKA	UKA	UKA	UKA	UKA
	4	HTO	HTO	HTO	HTO	HTO	UKA	UKA	UKA	UKA	UKA	UKA

### 1.4 Soft tissue laxity

**Fig. 4:**
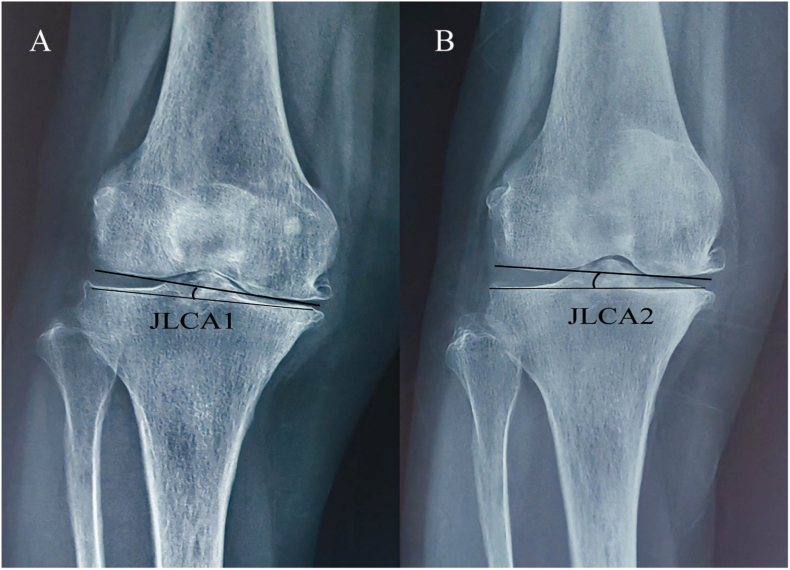
The disparity in JLCA between the supine (JLCA2) and upright (JLCA1) X-ray films is denoted as ΔJLCA; Joint line convergence angle (JLCA).

**Fig. 5:**
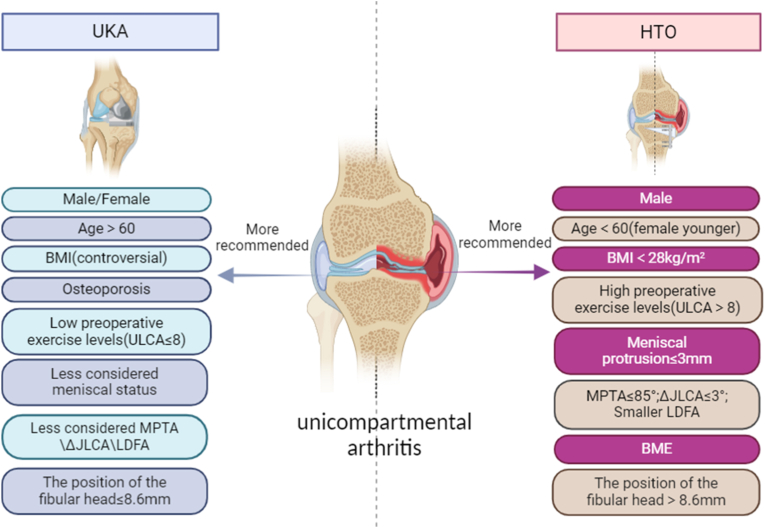
Factors influencing the choice of HTO or UKA treatment for patients with unicompartmental arthritis. Medial proximal tibial angle (MPTA); Joint line convergence angle (JLCA); Lateral distal femoral angle (LDFA); Bone marrow edema (BME).

Moreover, joint soft tissue laxity is closely tied to the prognosis of HTO surgery [40]. The most common method to quantify soft tissue laxity is through evaluating the joint line convergence angle (JLCA). Large changes in JLCA are linked to overcorrection [41,42]. The study revealed that latent medial laxity, calculated as the difference between JLCA on weight-bearing standing radiographs and JLCA on stress radiographs, was positively correlated with postoperative JLCA and correction angle. For each 1° increase in latent medial laxity, the postoperative change in JLCA increased by 0.6°, and for each 1° increase in correction angle, the postoperative change in JLCA increased by 0.2° [43]. A study conducted by Sang-Yeon So et al. revealed that the disparity in JLCA between supine radiographs and standing radiographs (ΔJLCA) is the key preoperative factor for predicting the variation in coronal correction after HTO and should be factored into the expected preoperative correction angle. Subtracting ΔJLCA can lead to a more optimal postoperative correction angle. These two methods of predicting overcorrection after HTO surgery demonstrate that preoperative JLCA is a significant predictor of postoperative efficacy. Patients with a preoperative ΔJLCA that is too large may not be suitable candidates for HTO surgery. For UKA, studies have indicated that a high medial tibial joint line (calculated as the thickness of the polyethylene pad and tibial tray minus the osteotomy amount and saw blade thickness) can negatively affect surgical outcomes [44]. However, no studies have elaborated on the relationship between JLCA and prognosis of UKA, highlighting the need for further research in this area. The JLCA measurement method entails determining the angle between the two joint tangents of the distal femur and the proximal tibia. These values are considered positive when the angle's apex is medial (varus) and negative when it is lateral (valgus). The disparity in JLCA between the supine and upright X-ray films is denoted as ΔJLCA (Fig. 4) [45] (see Fig. 5).

This indicates that latent medial laxity is the critical factor influencing postoperative JLCA changes and plays a crucial role in the decision-making process regarding the choice of HTO surgery for treatment. Some studies have also suggested that patients with significant OA and accompanying flexion spasm, necessitating extensive correction, may experience an increase in posterior tibial slope following HTO. This increase in posterior tibial slope could lead to anterior displacement of the tibia relative to the femur, thereby increasing tension on the anterior cruciate ligament and ultimately exacerbating the progression of OA [46,47].

### 1.5 Preoperative bone marrow edema, spontaneous insufficiency fracture of the knee and osteoporosis

Bone marrow edema (BME) in the subchondral bone is associated with OA. The most common cause of BME is repeated microfractures and subsequent vascular changes in the subchondral bone. BME is a common source of pain and joint changes, and understanding it is crucial for developing strategies to manage the natural progression of OA [48]. A study with a 10-year follow-up revealed a significant positive correlation between the severity of preoperative BME and postoperative pain after UKA [49]. Furthermore, several studies have researched the impact of preoperative BME on postoperative pain after UKA, consistently indicating that patients with preoperative BME experience significant pain following UKA surgery [50]. However, in patients treated with HTO surgery, some studies have revealed no significant correlation between the preoperative BME and the prognosis after HTO surgery. Also, patients have reported substantial improvements in pain and joint function following the HTO surgery [51].

Spontaneous insufficiency fracture of the knee (SIFK), previously known as spontaneous osteonecrosis of the knee (SONK), commonly occurs in the medial femoral condyle [52,53]. This condition, characterized by joint pain, has an unknown cause and can progress to collapse in severe cases. The primary imaging features include subchondral fractures and BME. Arthritis and varus deformity are significant factors contributing to its progression [54]. The primary surgical treatments for unilateral compartment OA associated with SIFK are UKA and HTO [55]. Therefore, the choice of treatment options for such patients warrants further study. A prospective study showed that, for SIFK, patients who underwent HTO experienced better improvement in clinical symptoms compared to those who received non-surgical treatment. Additionally, imaging revealed a significant reduction in edema around the lesion in the HTO group, although the necrosis area was not significantly different from that of the non-surgical treatment group [56]. Further studies have indicated that combining mosaicplasty with HTO can further reduce BME and improve patient prognosis [57]. Regarding the treatment with UKA, a retrospective study indicated that it provides significant improvement in clinical symptoms [58]. However, further research in the future is necessary to compare the efficacy of UKA with other surgical or non-surgical treatments. Some studies have also identified osteoporosis as a risk factor for requiring subsequent revision surgery in HTO patients [59]. However, preoperative bone density does not affect the prognosis of tibial component migration or pain after UKA surgery [60]. Therefore, UKA surgery is more recommended for patients with preoperative osteoporosis.

### 1.6 Basic preoperative demographic data: gender, age, and weight

#### 1.6.1 Gender

Gender is closely related to the progression of OA and impacts recovery after surgery. A Korean retrospective study found that risk factors for revision surgery in HTO patients include being over 60, female, and having comorbidities such as diabetes, osteoporosis, and hyperlipidemia [59]. A cohort study with a follow-up period of over six years confirmed the relationship between gender and HTO prognosis. This study included 245 patients and found that women who underwent HTO surgery were more likely to need joint replacement than men and had lower survival rates at 5 and 10 years [61].

However, a 5-year follow-up study found no statistical relationship between gender and prognosis after UKA surgery. There were no significant differences in imaging results, prosthesis positioning, or other factors [62]. These studies suggest that gender may be an important consideration in treatment selection for patients who are candidates for both HTO and UKA.

#### 1.6.2 Age

A prospective study of 643 patients found that advancing age is a risk factor for requiring a repeat TKA after HTO [63]. Cheng Jin et al. also found that being 65 years or older is a risk factor for poor prognosis following HTO surgery [64]. A 2021 retrospective study from South Korea examined the relationship between age and the prognosis of HTO. The study found that patients under 60 years old had lower revision and complication rates compared to those over 60 years old [65]. A 20-year follow-up study also showed that the prognosis after HTO was better in patients under 55 years old [66].

However, Song JH et al. have divided HTO patients into older and younger groups using a median age of 55 years. By matching patients in each group based on preoperative arthroscopic assessments of cartilage status, these studies found that the prognosis of HTO surgery is influenced by cartilage status, regardless of age [67].

Research on the relationship between age and the prognosis of UKA surgery has shown that age is not a significant factor in poorer outcomes or higher revision rates. A meta-analysis indicated that the age of patients undergoing UKA surgery does not statistically affect prognosis, meaning age is not an absolute contraindication for UKA [68]. A 10-year follow-up study categorized patients into groups aged over or under 55 years old, revealing no statistical difference in prognosis after UKA [69]. Graham S. Goh et al. further confirmed that patient age does not impact the prognosis of UKA. They achieved this by matching elderly patients over 80 with those aged 65–74 who underwent UKA [70].

Therefore, we currently recommend that patients who require a higher level of physical activity after surgery consider HTO if they are under 60 years old. For those over 60, UKA is recommended. Since women undergoing HTO are more likely to have a poor prognosis, the recommended age range for female patients should be adjusted accordingly when selecting treatment options.

#### 1.6.3 Weight

A prospective study found that a high BMI is closely associated with an increased risk of requiring repeat TKA surgery after HTO [63]. A 10-year follow-up study further confirmed this finding, showing that a higher BMI was linked to poorer joint functional recovery and increased joint pain after HTO [71]. Some studies have also noted that an excessive BMI primarily impacts mid-term outcomes for patients following HTO surgery [72]. Haichuan Guo et al. found that a BMI≥28 kg/m^2^ is an independent risk factor for deep vein thrombosis following HTO surgery [73].

There is currently some controversy regarding the relationship between BMI and the prognosis of UKA. Studies have shown that obesity does not result in a worse prognosis for UKA [74,75]. A retrospective study validated this finding, indicating that obese patients undergoing fixed-bearing lateral UKA can achieve satisfactory clinical outcomes [76]. A 10-year prospective study emphasized that obesity does not result in a worse prognosis for UKA, suggesting that obesity should not be viewed as an absolute contraindication for the UKA [77].

However, a prospective study pointed out that although obese patients may also experience some improvements following UKA surgery compared to preoperative situation, they typically exhibit poorer long-term joint function and an increased risk of postoperative revision at the 10-year mark [78]. A retrospective study also corroborated this perspective, highlighting that the early revision rate after UKA in patients with a BMI>40 kg/m^2^ was five times higher than that in the control group [79]. This suggests that while the impact of obesity on UKA surgery might be relatively mild in the early postoperative period, it could significantly affect long-term outcomes and the rate of surgical revisions. Further high-quality, long-term studies are needed to explore this hypothesis.

Therefore, Currently, when other preoperative factors are similar, we recommend UKA surgery for obese patients (BMI>28 kg/m^2^) and HTO surgery for those with a BMI<28 kg/m^2^.

## 2 Disscussion

By correlating preoperative factors with postoperative prognosis, new criteria for indications are established to guide specific groups of individuals in choosing between UKA or HTO.

Previous studies have suggested that patients who are older than 55 years old, have unilateral compartment OA, experience bone-to-bone meniscus grinding, exhibit a varus deformity (TBVA) of less than 5° [80], engage in moderate exercise intensity, have a flexion deformity of less than 10° and maintain intact ligament function, are more suitable candidates for UKA surgery [81,82]. Patients younger than 65 years old, with medial OA, partial cartilage damage, TBVA greater than 5° [83], high exercise intensity, flexion deformity less than 20°, and intact ligaments are more suitable candidates for HTO surgery [5,84].

As mentioned earlier, current research indicates that patient gender does not influence the prognosis of UKA, while men tend to be more suitable candidates for HTO. Patients under 60 years old are generally more suitable for HTO surgery, while those over 60 are more often recommended for UKA treatment [65,85]. HTO surgery offers advantages for unilateral OA patients with BMI<28 kg/m^2^, high preoperative exercise intensity, low meniscal extrusion, small relevant angles (KJLO, LDFA, JLCA), fibular head height more than 8.6 mm, or bone marrow edema. This suggests the need to update traditional surgical indications and contraindications to offer patients more appropriate treatment options, particularly those who meet criteria for both traditional UKA and HTO surgery. Therefore, this article analyzes several aspects of preoperative patient factors, including exercise intensity, meniscal status, anatomical structure, soft tissue laxity, bone marrow edema, osteoporosis and patient's overall condition, to provide more tailored treatment recommendations for individuals with unicompartmental OA (Table 1). However, there is still some controversy regarding the preoperative factors influencing the prognosis of UKA or HTO surgery, as well as a lack of specific quantification standards. Therefore, additional high-quality studies are needed to further explore these aspects and establish more comprehensive criteria for UKA and HTO surgery indications.

**Fig. 6:**
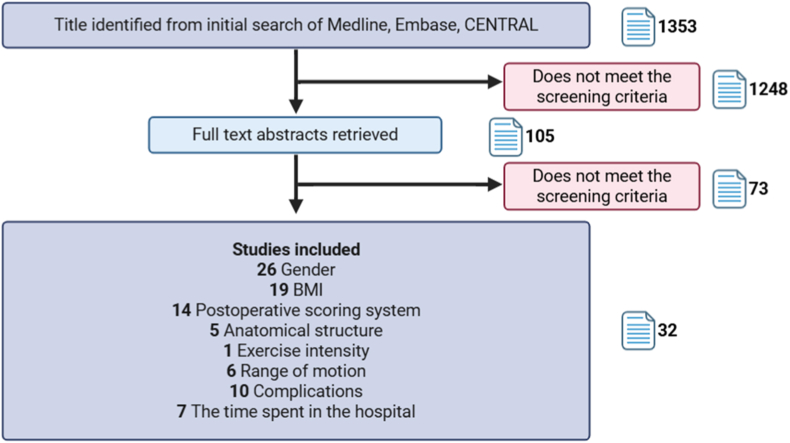
Flowchart of the study selection process. Data were derived from 32 studies, with some contributing to analyses across multiple domains. Body Mass Index (BMI).

**Fig. 7:**
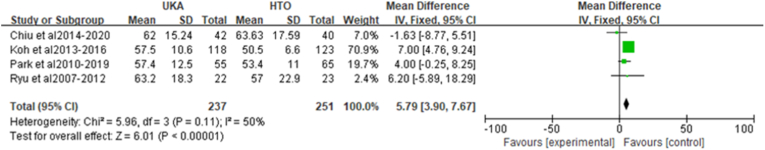
A forest plot comparing the preoperative WOMAC scores of patients undergoing UKA and HTO in the subgroup with favorable postoperative recovery.

To provide a stronger theoretical basis for selecting the most favorable surgical plan, we conducted a meta-analysis of existing studies. A total of 32 eligible studies were included and categorized into two groups according to postoperative knee joint-related scores: Group 1, the satisfactory recovery group (defined as a postoperative KSS >120, HSS >60, WOMAC <28, or KOOS >60) [9,[86], [87], [88], [89], [90], [91], [92], [93], [94]], and Group 2, the unsatisfactory recovery group (defined as a postoperative KSS ≤120, HSS ≤60, WOMAC ≥28, or KOOS ≤60) (Fig. 6) [11,95]. Analysis of the two groups showed that, among patients with satisfactory postoperative recovery, those who underwent UKA had a significantly higher preoperative WOMAC score compared with those who underwent HTO (5.79 [3.90–7.67]) (Fig. 7). In addition, UKA patients had a significantly smaller preoperative flexion contracture angle (−1.43 [−2.40 ∼ −0.46]), a higher preoperative UCLA score (0.20 [0.02–0.38]), a smaller preoperative tibial slope angle (−5.87 [−7.15 ∼ −4.59]), and a greater preoperative MPTA (0.90 [0.22–1.58]) than HTO patients. However, there were no significant differences between the two groups in terms of preoperative BMI or flexion angle. Among patients with suboptimal postoperative recovery, no statistically significant differences were observed in preoperative BMI, WOMAC scores, or flexion angle between those undergoing UKA and HTO (detailed analytic methods and additional results are provided in Supplementary Material). Both UKA and HTO remain viable options for the treatment of isolated unicompartmental osteoarthritis. By comparing preoperative factors associated with surgical efficacy, this study identified significant differences in preoperative WOMAC scores, flexion contracture angle, UCLA scores, tibial posterior slope angle, and MPTA between patients who underwent UKA and those who underwent HTO, specifically among individuals with favorable postoperative recovery. These findings may help guide the preoperative selection of the most appropriate surgical option for individual patients. Detailed analysis methods and additional results are provided in the Supplementary Material.

Enhancing the surgical process based on preoperative factors can contribute to further improving surgical outcomes.

In addition to choosing different surgical methods for treatment based on the patient's preoperative condition, doctors can also adapt the surgical approach according to preoperative factors to optimize the patient's prognosis. As mentioned earlier, the study revealed a positive correlation between the degree of latent soft tissue laxity (ΔJLCA) measured before surgery and postoperative JLCA and correction angle [43]. Hence, in the future, adjusting the correction angle appropriately based on preoperative JLCA may ensure that the final correction angle aligns with expectations, thus preventing over-correction and enhancing patient prognosis.

Additionally, some studies have indicated that patients with a high fibular head often exhibit a small lateral tibial condyle, increasing the likelihood of developing lateral hinge fractures following HTO surgery [31]. Therefore, this suggests that we can refine the osteotomy plan for such patients based on their preoperative anatomy to mitigate the risk of poor prognosis.

HTO tends to elevate the tibial slope, a phenomenon that heightens stress on the anterior cruciate ligament (ACL) and poses a risk factor for both primary and secondary ACL injuries [47,96]. The tibial slope can be decreased by internally rotating the osteotomy to form an anterolateral hinge. Therefore, the choice of osteotomy method should be based on the ACL status prior to HTO surgery to enhance surgical outcomes [97,98].

Some studies have also noted that while patients with severe preoperative flexion contractures may achieve better mid-term survival rates following UKA surgery, they may still experience residual flexion contractures and limited joint range of motion [99]. Further research has indicated that, compared to TKA, although flexion contracture may persist after UKA, postoperative movement and joint function recovery tend to be superior [100].

Some studies suggest that elevating the medial tibial joint line (calculated as the thickness of the polyethylene pad and tibial tray minus the osteotomy amount and saw blade thickness) during UKA surgery may hinder the improvement of joint motion angles. It is recommended to avoid elevating the medial tibial joint line by more than 5 mm during UKA procedures to mitigate the extent of postoperative flexion contracture [44].

For HTO surgery, some studies have indicated that performing notchplasty during HTO can alleviate flexion contracture [101]. Hence, for patients with severe preoperative flexion contracture, exploring potential enhancements in surgical techniques may lead to improved postoperative outcomes. In addition, we can utilize new materials, such as polyitaconate-based hydrogel (PICGI), to better prevent infection in surgical incisions and promote tissue healing, thereby accelerating postoperative recovery [102].

Consideration should also be given to the impact of preoperative factors on surgical complications.

Current research indicates that thorough evaluation of patients' preoperative conditions can not only enhance postoperative joint recovery but also offer insights into potential postoperative complications. A retrospective study involving 8353 patients revealed that individuals who underwent knee arthroscopy within 2 years prior to UKA were more likely to require postoperative TKA and experience aseptic implant loosening resulting in UKA failure, compared to those who did not undergo knee arthroscopy [103].

For patients with rheumatoid arthritis (RA), a study found that those who underwent UKA did not have an increased risk of subsequent revision to TKA compared to patients without RA [104]. For patients with preoperative RA, except for a 1977 study indicating that HTO surgery can improve the clinical symptoms of RA patients [105], no studies have compared the prognosis of RA patients with that of non-RA patients undergoing HTO surgery in recent years. Therefore, it is important to conduct further research to determine the clinical benefits for patients with autoimmune diseases undergoing UKA or HTO. Additionally, studies have shown that patients with diabetes have a higher incidence of deep vein thrombosis within three months after UKA surgery compared to patients without diabetes [106,107]. Some studies have also identified diabetes as a risk factor for postoperative revision following HTO surgery [59]. However, no studies have compared the prognosis of diabetic patients undergoing UKA versus HTO surgery. A 15-year study found that smoking was a demographic variable associated with post-HTO complications, with the most common issues being stiffness, superficial wound infection or dehiscence, and deep infection [108]. Generally, investigating preoperative factors can help anticipate and avoid potential postoperative complications, providing more informed treatment options for patients who meet the criteria for both traditional HTO and UKA.

## Author contribution statement

Jianbin Guo and Zhibo Liu analysed the data and wrote the manuscript. Yong Ding edited the manuscript. All authors reviewed the data, approved the final version of the manuscript, and agreed to take full responsibility for the work.

## Declaration of generative AI in the scientific writing statement

We did not use any AI tools in the preparation of this article. All authors contributed to the editing and review of the manuscript and take full responsibility for the final published content.

## Declaration of competing interest

The authors declare no conflict of interest. The outside source of funds was not involved in data collection, data analysis, preparation or editing of the manuscript.
