# Supplementary material for: How to choose treatment for patients who are eligible for both unicompartmental knee arthroplasty and high tibial osteotomy?
Source: J Orthop Translat. 2025 Dec 22;56:101023. doi: 10.1016/j.jot.2025.10.015 (PMC12988496; doi:10.1016/j.jot.2025.10.015)
Supplement: Multimedia component 1 [file mmc1.docx]

**Methods**

Data extraction

This study aimed to provide a clear and comprehensive summary of published data on unicompartmental knee arthroplasty (UKA) and high tibial osteotomy (HTO), focusing on preoperative factors that are critical for patients and clinicians in making informed decisions. A systematic search of Medline, Embase, and the Cochrane Central Register of Controlled Trials (CENTRAL) was conducted for studies published between January 1, 2000, and August 1, 2025. We searched for studies published within the past 25 years that directly compared UKA and HTO in adult patients (≥18 years). Eligible study designs included randomized controlled trials, retrospective analyses of large national or multicenter databases or joint registries, and large cohort studies. Studies focusing exclusively on isolated patellofemoral or isolated lateral compartment osteoarthritis were excluded. Conference abstracts and case reports were excluded. To minimize overestimation of results, only studies with at least 20 participants were considered. A minimum follow-up of 30 days was required for studies assessing adverse events, and at least 6 months for those evaluating clinical outcomes. Studies with insufficient data for statistical analysis were excluded. Non-English studies were translated and included whenever possible. Study selection was performed independently by two authors (LZB and GJB) based on titles and abstracts, followed by full-text review. Discrepancies were resolved through discussion with a third author (DY).

Data were extracted using a standardized form. Two authors (LZB and GJB) independently extracted information from all included studies. When available, recorded variables included the principal investigator’s name, research period, sample size, and patient demographics (age and sex). Data on primary outcome measures and adverse events were also collected.

**Table 1.** Summary of studies included and their key features.

Risk of bias

Risk of bias in randomized controlled trials was assessed using the Cochrane collaboration’s tool, evaluating random sequence generation, allocation concealment, blinding of participants and outcomes, and attrition bias, each categorized as low, unclear, or high risk.

**Table 2.** Risk of bias table for RCTs using Cochrane collaboration’s tool. (√= clear documentation that the study meets this requirement, **U** = unclear based on the publication, X = no evidence that the requirement was met.)

Data Analysis

This study was conducted in accordance with the MOOSE (Meta-analysis of Observational Studies in Epidemiology) and PRISMA (Preferred Reporting Items for Systematic Reviews and Meta-Analyses) guidelines. Inverse variance–weighted analysis was used to calculate overall summary estimates. Individual relative risk estimates and pooled estimates were presented in forest plots. Heterogeneity was assessed using the I² statistic and the Q test. A p-value less than 0.05 was considered statistically significant.

**Result**


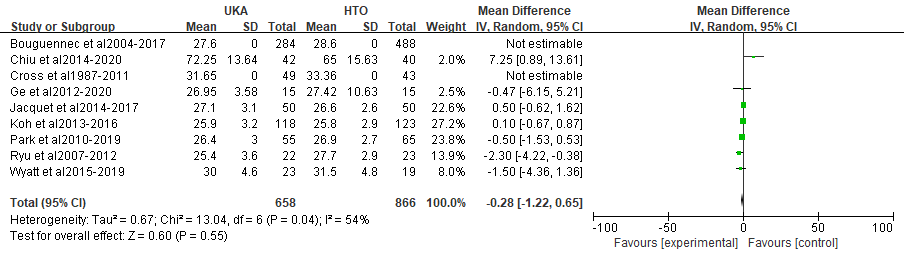
**Figure 1.** The forest plot comparing preoperative BMI between UKA and HTO patients in the subgroup with favorable postoperative recovery showed no significant difference.


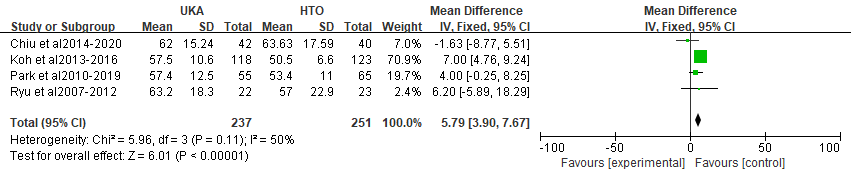


**Figure 2.** The forest plot demonstrated that, among patients with favorable postoperative recovery, preoperative WOMAC scores were significantly higher in those who underwent UKA compared with those who underwent HTO.


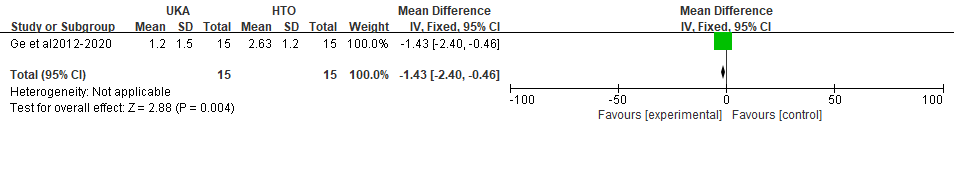


**Figure 3.** The forest plot showed that, among patients with favorable postoperative recovery, the preoperative flexion contracture angle was significantly smaller in those who underwent UKA compared with HTO.


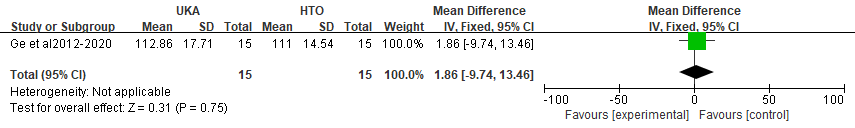
 **Figure 4.** The forest plot showed no significant difference in preoperative flexion angle between UKA and HTO patients among those with favorable postoperative recovery.


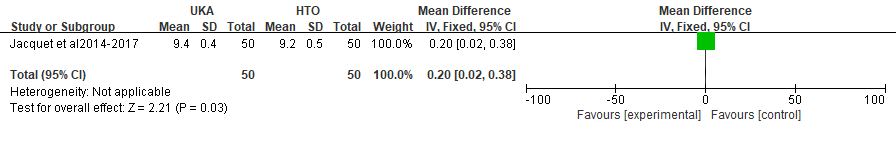


**Figure 5.** The forest plot showed that, among patients with favorable postoperative recovery, preoperative UCLA scores were significantly higher in those who underwent UKA compared with HTO.


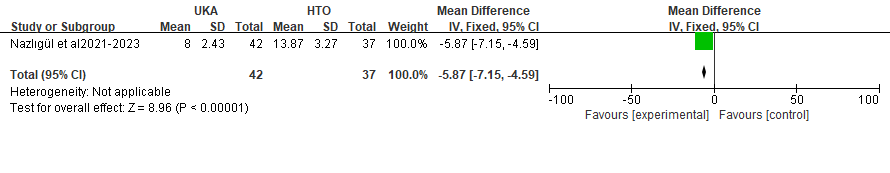


**Figure 6.** The forest plot showed that, among patients with favorable postoperative recovery, the preoperative tibial slope angle was significantly smaller in those who underwent UKA compared with HTO.


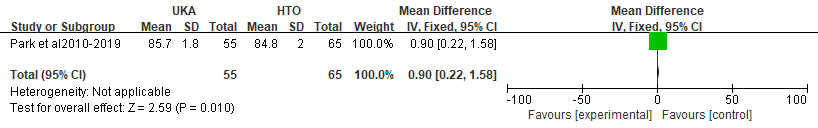


**Figure 7.** The forest plot showed that, among patients with favorable postoperative recovery, preoperative MPTA was significantly greater in those who underwent UKA compared with HTO.


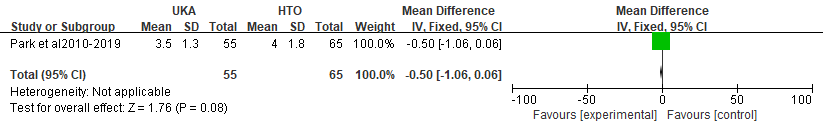


**Figure 8.** The forest plot showed no significant difference in preoperative JLCA between UKA and HTO patients among those with favorable postoperative recovery.


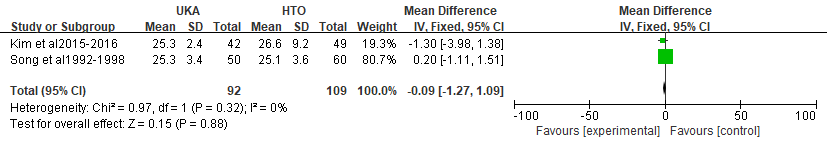
 **Figure 9.** The forest plot showed no significant difference in preoperative BMI between UKA and HTO patients among those with suboptimal postoperative recovery.


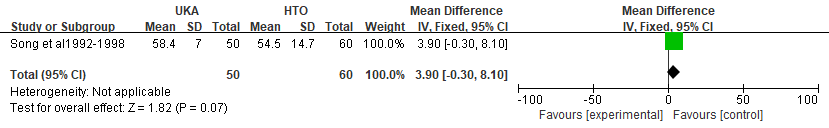


**Figure 10.** The forest plot showed no significant difference in preoperative WOMAC scores between UKA and HTO patients among those with suboptimal postoperative recovery.


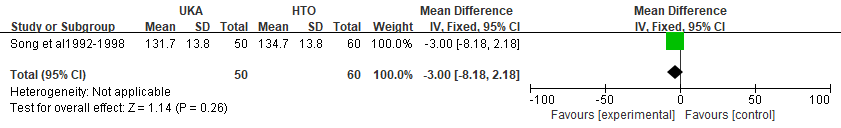


**Figure 11.** The forest plot showed no significant difference in preoperative flexion angle between UKA and HTO patients among those with suboptimal postoperative recovery.
